# An Informatics Bridge to Improve the Design and Efficiency of Phase I Clinical Trials for Anticancer Drug Combinations

**DOI:** 10.1158/2767-9764.CRC-22-0160

**Published:** 2022-09-06

**Authors:** Lei Wang, Lai Wei, Shijun Zhang, Lijun Cheng, Aditi Shendre, Williams Carson, James L. Chen, Dwight Owen, Megan Gregory, Lang Li

**Affiliations:** 1 Department of Biomedical Informatics, The Ohio State University College of Medicine, Columbus, Ohio.; 2 Division of Surgical Oncology, The Arthur G. James Cancer Hospital and Richard J. Solove Research Institute, Comprehensive Cancer Center, The Ohio State University, Columbus, Ohio.; 3 Department of Surgery, Comprehensive Cancer Center, The Ohio State University, Columbus, Ohio.; 4 Division of Medical Oncology, Department of Internal Medicine, The Ohio State University Comprehensive Cancer Center, Columbus, Ohio.

## Abstract

In this study, we summarized critical databases of drug combination toxicity and pharmacokinetics. We further conducted a feasibility and utility study that demonstrates how different data sources can contribute to and assist phase I trial designs. Single-drug and drug combination toxicity and pharmacokinetic data were primarily reviewed from several databases. We focused on the MTD, dose-limiting toxicity (DLT), toxicity, and pharmacokinetic profiles. To demonstrate the feasibility and utility of these data sources in improving trial designs, phase I studies reported in ClincalTrials.gov from January 1, 2018 to December 31, 2018 were used as examples. We evaluated whether and how these studies could have been designed differently given toxicity and pharmacokinetic data. None of the existing pharmacokinetic and toxicity databases contain either MTD or DLT. Among 268 candidate trials, four drug combinations were studied in other phase I trials before 2018; 185 combinations had complete or partial information on drug interactions or overlapping toxicity, and 79 combinations did not have available information. Two drug combination trials were selected as case studies. The nivolumab-axitinib trial could have been designed as a dose deescalating study, and the vinorelbine-trastuzumab emtansine trial could have been designed with a lower dose of either drug. Public data sources contain significant knowledge of the drug combination phase I trial design. Some important data (MTD and DLT) are not available in existing databases but in the literature. Some phase I studies could have been designed more efficiently with additional preliminary data.

**Significance:**

Prior preclinical and clinical knowledge is critical for designing effective and efficient cancer drug combinatory trials. We reported results on the feasibility and utility of different informatics resources for contributing to and assisting phase I trial designs based on our designed classification approach. We also found that public data sources contained significant knowledge for drug combination phase I trial design, but some critical data elements (MTD and DLT) were missing.

## Introduction

Multidrug therapies targeting multiple disease mechanisms are more efficacious in most patients with cancer than single-drug therapies. In the meantime, more drug combinations have undergone clinical trials for cancer. ClinicalTrial.gov data have demonstrated that the number of cancer drug combination trials has increased from 445 in 2007 to 912 in 2019. However, many clinical trials have failed in this regard. A recent study of pharmaceutical drug development between January 1, 2000 and October 31, 2015 (1) reported failures of 7,346 phase I (58%), 4,396 phase II (35%), and 797 phase III (7%) cancer trials. These numbers highlight the failure of phase I trials as the biggest challenge in the development of cancer drugs for clinical use.

A phase I trial is a dose-finding study that investigates the safety and tolerability of a drug or drug combination (2). Its goal is to determine the MTD, optimal dose level, and schedule to recommend for evaluation in a phase II trial of an experimental drug or drug combination in a target patient population(s; ref. 3). “3+3” design continues to be commonly used in phase I trials. Properly designed phase I trials should minimize patient exposure to severe or life-threatening adverse drug events (ADE) and treat more patients at the optimum dose that produces a therapeutic effect while ensuring safety. In the last few decades, the statistical community has extensively developed many innovative phase I trial designs to improve operating characteristics compared with the standard “3+3” design (4–6). However, regardless of how powerful statistical designs can be, their success relies on accurate prior knowledge of single-drug or drug combination toxicities, the period of DLT evaluation, the rate of DLT at the MTD, and potential pharmacokinetic drug–drug interactions (DDI).

Table 1 delineates the various data sources that archive a large amount of information regarding the pharmacokinetics and toxicity of drugs. After reviewing these publicly available data sources (see Supplementary Data S1), we found several primary challenges for prior evidence knowledge query and collection for drug combination phase I trials. First, structured pharmacokinetic and toxicity data were distributed in different databases and were not combined. Second, important data elements, such as MTDs and DLTs of single drugs and drug combinations, are not collected in any databases but in the scientific literature. Third and most importantly, we have limited knowledge on whether and how these data sources can properly assist us in designing drug combination phase I trials.

**TABLE 1:** Summary of data sources and their providing information

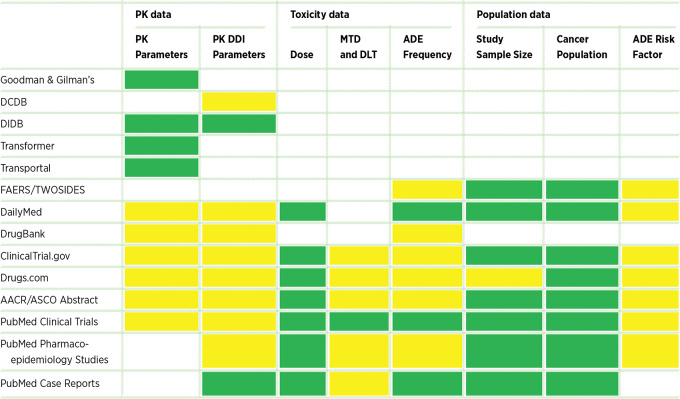								

NOTE: Green: Database have complete information Yellow: Database have partial information.

Abbreviations: ADE: adverse drug events; DCDB: drug combination database; DDI: drug–drug interaction; DIDB: The Drug Interaction Database; DLT, dose-limiting toxicity; FAERS: Search Results FDA Adverse Event Reporting System; FM database: the fraction of metabolism database; Goodman & Gilman's: Goodman & Gilman's The Pharmacological Basis of Therapeutics; MTD, maximum tolerance dose; PK, pharmacokinetic.

In this study, we designed a classification approach, which integrated pharmacokinetic DDI and toxicity evidence and knowledge collected from various data sources, for drug combination phase I clinical trial. Meanwhile, we examined the process and demonstrated the feasibility of a cancer drug combination phase I clinical trial design based on our proposed classification approach.

## Materials and Methods

### A Feasibility and Utility Study of Data Sources in Assisting Phase I Cancer Drug Combination Clinical Trial Designs

To demonstrate the feasibility and utility of data sources for designing cancer drug combination phase I trials, we evaluated the knowledge coverage and gaps in pharmacokinetic and toxicity data from a number of data sources.

#### Candidate Phase I Trial Selection

We targeted phase I or IB cancer drug combination clinical trials registered in ClinicalTrials.gov from January 1 to December 31, 2018. Relevant data (e.g., clinical trial titles and interventions) were extracted and analyzed from the Aggregate Analysis of ClinicalTrials.gov (AACT), a publicly available relational database containing all information in ClinicalTrials.gov. In our analysis, we included trials with only a two-drug intervention owing to the lack of available DDI data sources for drug combinations that involved more than two drugs. We also limited that the two-drug intervention could involve either small-molecule cancer drugs or therapeutic proteins. These two-drug interventions were further reviewed manually to remove trials in which the inventions contained radiotherapy and cell-based therapy. This ensured that all trial candidates involved only individual small-molecule drugs or mAbs. Finally, DrugBank ID (7) was used to normalize all drug names. Recognizing that a number of investigational drugs had no generic or brand names, their reported drug names in ClinicalTrials.gov were retained for further identification.

#### Drug Combination Pharmacokinetics and Toxicity Data Review and Preparation

Single-drug toxicity data and their corresponding frequencies were manually retrieved from the DailyMed website and SIDER (version 4.1, released on October 21, 2015, RRID:SCR_004321; ref. 8). Then, toxicity data were mapped to proper MedDRA preferred terms (PT) to check the overlapping toxicities between the two drugs. In the current study, an identical PT shared by the two drugs was considered an overlapping toxicity.

The pharmacokinetic DDI evidence was reviewed and collected mainly from DrugBank (version 5.0.10, RRID:SCR_002700; ref. 7) and the published literature. We chose an old version of the DrugBank database to ensure that all pharmacokinetic DDI data had been published before phase I trial candidates began. The combination of a small-molecule drug and a mAb or two mAbs was considered as a nonpotential pharmacokinetic DDI pair based on the mechanism of pharmacokinetic DDI, because small-molecule drugs are typically metabolized through CYP450s and other phase II metabolic enzymes in the human liver, whereas mAbs are mostly degraded in targeted tissues other than the liver. Because all the evidence was derived from publicly available machine-readable data sources, we did not conduct a validation step in the current study.

#### Evidence Strength Annotation Schemes for Drug Combination Toxicity and Pharmacokinetics

##### Annotation of Drug-toxicity Data

**FIGURE 1:**
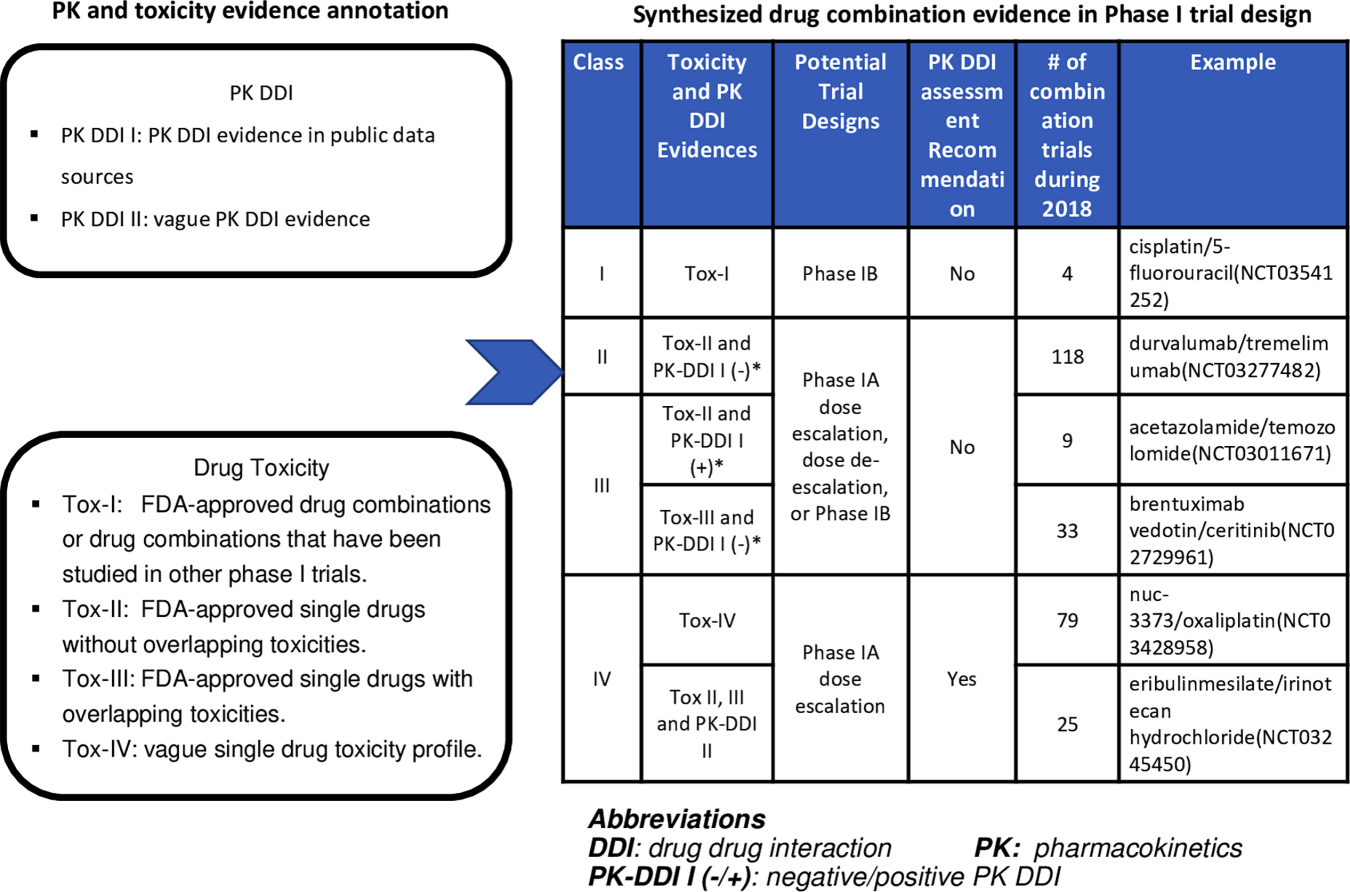
Classification of phase I or IB cancer drug combination clinical trials registered in ClinicalTrials.gov from January 1 through December 31, 2018 based on their information completeness on pharmacokinetic interactions and toxicity.

Evidence of the toxicity of drug combinations can be broadly categorized into four types (Fig. 1).

- Tox-I evidence: FDA-approved drug combinations or combinations with published phase I clinical trial results where no severe adverse events were observed (successful phase I trial results) were considered to have Tox-I evidence.

Drug combinations consisted of FDA-approved drugs that have public successful phase I trial results for the individual components are considered Tox-II or Tox-III.

- Tox-II evidence: No overlapping toxicity between individual drugs in the combination fulfills Tox-II criteria.
- Tox-III indicated that overlapping toxicities existed.
- For Tox-IV scenario, at least one drug lacks sufficient toxicity data or toxicity data are reported in case studies or the FDA Adverse Event Reporting System (FAERS) but not in clinical trials.

##### Annotation of Pharmacokinetic DDI Data

Pharmacokinetic DDI evidence was categorized into two types.

- Pharmacokinetic DDI level I evidence in this study consisted of both positive and negative evidence. The DrugBank pharmacokinetic DDI evidence or published literature was considered positive evidence. Combinations of a small-molecule drug and a mAb or two mAbs were considered negative (described in Drug Combination Pharmacokinetics and Toxicity Data Review and Preparation).
- Pharmacokinetic DDI II evidence in this study refers to combinations of two small-molecule drugs that have no pharmacokinetic DDI information in the DrugBank and published literature.

#### Joint Drug Combination Toxicity and Pharmacokinetic Evidence Annotation Scheme and Its Implication for Cancer Drug Combination Phase I Trial Design

On the basis of the above evidence strength annotation schemes, we designed a classification approach that synthesize drug combination toxicity and pharmacokinetic evidence for phase I clinical trials. Their implications for cancer drug combination phase I trial design are summarized in the following (Fig. 1).

- Class I: A drug combination that has already been approved by the FDA (Tox-I), in which no pharmacokinetic DDI evidence is needed. The toxicity of these drugs has been adequately evaluated under FDA-approved indications and cancer patient populations. If this drug combination is tested in a new population of cancer patients, a phase IB study could be a viable option.
- Class II: Two drugs have negative pharmacokinetic DDI evidence (PK-DDI I) and no overlapping of DLT (Tox-II). In such cases, a dose deescalation phase I study could be an option.
- Class III: Two drugs have either overlapping DLT (Tox-III) and negative PK-DDI or nonoverlapping DLT (Tox-II) and positive PK-DDI. In this case, the starting dose of the drug combination should be carefully chosen, and a dose-escalation phase I trial could be an option.
- Class IV: At least one drug is not approved by the FDA. Both drugs have limited publicly available pharmacokinetic and toxicity data. Class IV represents the weakest evidence regarding the toxicity and pharmacokinetic DDI of drug combinations. It almost warrants a dose-escalation trial with carefully selected doses and may also require a drug combination pharmacokinetic study.

### Special Case Studies of Two Phase I Drug Combination Studies

Two case studies were selected to demonstrate whether and how drug combination studies could have been designed differently, given prior toxicity and pharmacokinetic knowledge. The screening criteria included (i) the post date or start date of selected cases was January 1 through December 31, 2018; (ii) the trial had a two-drug combination; and (iii) the original design document should be provided in the databases.

**FIGURE 2:**
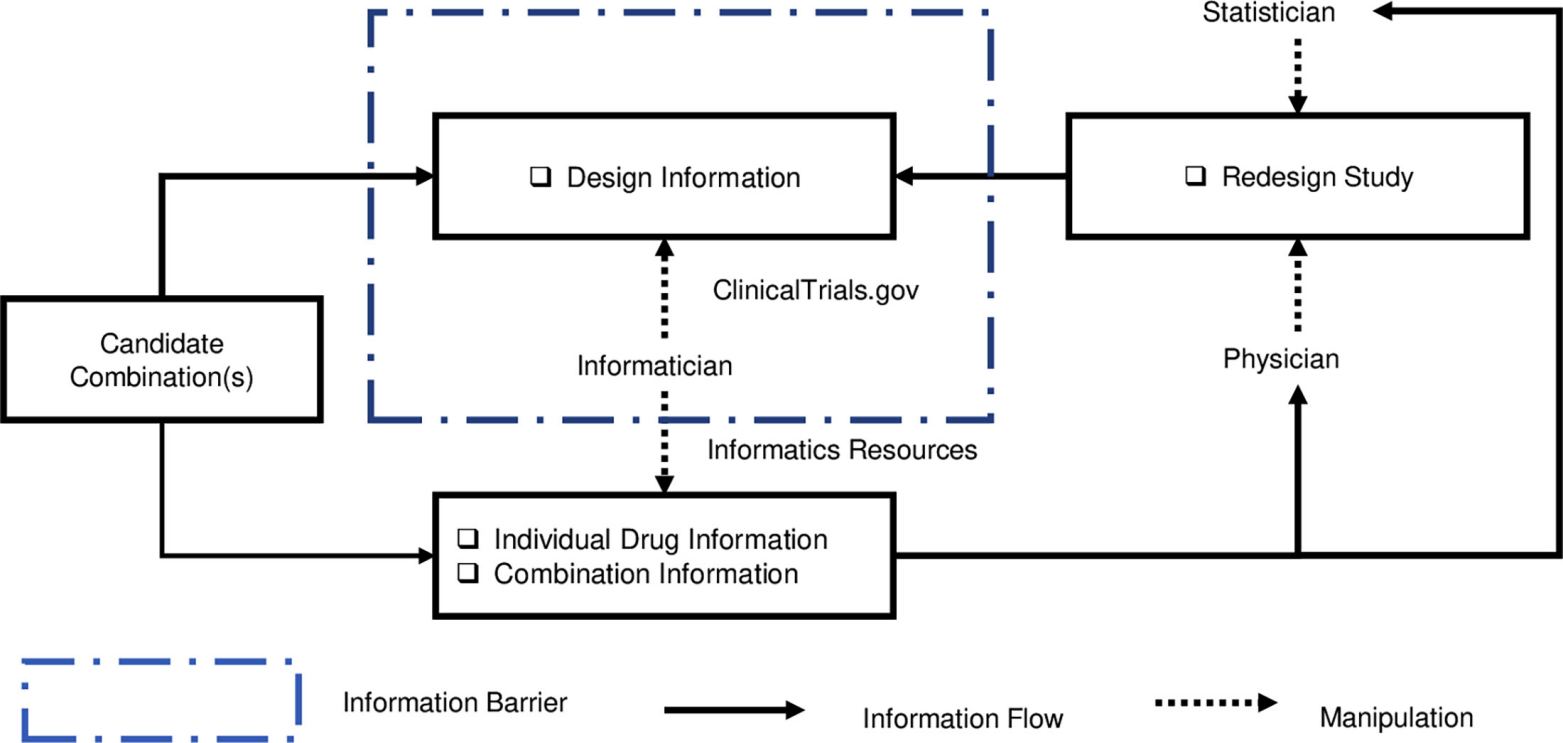
Overview of the utilization of prior knowledge to redesign phase I trials for two cases of drug combinations. The informatician first extracted the original study design for the candidate drug combinations from ClinicalTrials.gov and ensured blinding of this information to physicians and statisticians to avoid bias and inference. The informatician then integrated the background information from the original protocol and all additional pharmacokinetic and toxicity data from informatics resources involving individual drug and combination in the candidate combinations. And individual drug and combination information will be sent to physicians and biostatisticians as prior knowledge for decision making on phase I trial redesign. Finally, the informatician will compare the prior knowledge-based design with the original study design.

The case study consisted of two stages. The physician, biostatistician, and informatician engaged in the study. In the knowledge preparation stage, the informatician first extracted the original study design for the candidate drug combinations from ClinicalTrials.gov and ensured blinding of this information to a physician and statistician to avoid implicit bias. The informatician then integrated the background information from the original protocol and all additional single-drug and drug combination pharmacokinetic and toxicity data from various sources (Fig. 2).

The original protocol provided the mechanism of action of the individual drugs. Additional pharmacokinetic data were collected from the Cancer Drug Fraction of Metabolism (FM) database (9), Drug-interaction Database (DIDB), University of California, San Francisco (UCSF)-Transportal (10), DrugBank (7), and Drugs.com. Toxicity data were extracted from drug labels. All extracted ADEs were mapped to proper MedDRA PT terms, and ADEs in the boxed warnings section and warns and precaution section of drug labels were defined as severe. Moreover, MTDs were retrieved from PubMed (RRID:SCR_004846) using the query terms: drug name AND (MTD OR P2RD). The availability of all data from public informatics sources was also assured before the first publication date of the candidate trials on ClinicalTrials.gov.

Then, all these data were shared with a physician and a statistician, who designed at least one drug combination phase I clinical trial. The physician and statistician conducted their phase I trial design for the same cancer drug combination, taking the background information (both from the original protocol and public data sources) into consideration. Finally, their designs were compared with those published in ClinicalTrial.gov.

### Data Availability

The authors confirm that the data supporting the findings of this study are available within the article and its Supplementary Materials and Methods.

## Results

### Coverage in Public Data Sources of Pharmacokinetics and Toxicity Information for Cancer Drug Combination Phase I Clinical Trials (January 1 Through December 31, 2018)

We identified 423 phase I cancer drug combination trials in ClinicalTrials.gov from January 1, 2018 to December 31, 2018. These trials assessed 487 unique drugs in a total of 496 combinations. Some trials have investigated multiple drug combinations in different treatment arms. After intervention identification and drug name normalization, 268 unique two-drug combinations involving 252 unique drugs (including both small-molecule drugs and therapeutic proteins) were evaluated using publicly available data sources for their pharmacokinetic and toxicity properties. Among these 252 individual drugs, 112 were identified as small-molecule drugs by DrugBank or published literature, whereas 40 drugs were identified as therapeutic proteins. Prior to 2018, there were no available data sources to identify the remaining drugs.

Among the 268 two-drug combinations, 77 consisted of two small-molecule drugs, and 53 combinations consisted of two therapeutic proteins. The remaining combines a small-molecule drug and therapeutic protein as an intervention. For pharmacokinetic DDI-positive evidence, we focused on two small-molecule drug combinations, based on the pharmacokinetic DDI annotation scheme. We identified nine combinations of pharmacokinetic DDI-positive evidence from DrugBank. In terms of toxicity evidence, 39 of the individual drugs had a toxicity profile in the SIDER database. In addition, we extracted the toxicity profiles of 32 individual drugs from their labels.

After reviewing all data sources and applying our annotation schemes for drug toxicity, pharmacokinetics, and joint evidence classification criteria. The following are some highlights of this study (Fig. 1; Supplementary Data S2).

- Class I: Four of the 268 drug combinations were approved or studied under other conditions or patient populations. These four drug combinations may not need phase IA trials, and a phase IB study could be adequate.
- Class II: A total of 82 drug combinations had adequate data showing that they had no overlapping toxicity and negative pharmacokinetic DDI evidence. Therefore, we may use single-drug toxicity and pharmacokinetic data to determine their combination doses, and a dose deescalation phase IA trial could be an option.
- Class III: We found nine drug combinations with nonoverlapping toxicity but positive pharmacokinetic DDI evidence, and 33 drug combinations with overlapping toxicity but negative pharmacokinetic DDI evidence. Consequently, these 42 drug combinations and their dose selection should be carefully considered. A dose-escalation phase IA trial may be an option.
- Class IV: A total of 79 drug combinations have at least one drug with vague toxicity data, and 61 drug combinations have neither vague pharmacokinetic DDI nor toxicity evidence. These drug combinations will definitely require dose-escalation phase IA trials and potential additional pharmacokinetic DDI studies.

### Case Studies for Cancer Drug Combination Phase I Clinical Trial Design

#### Case 1, Nivolumab and Axitinib

##### Original Study Design

A phase IA trial of nivolumab and axitinib (NCT03172754) was conducted in patients with advanced renal cell carcinoma in 2017. The nivolumab and axitinib combination was tested at three dose levels: (2 mg twice daily + 480 mg every 4 weeks), (3 mg twice daily + 480 mg every 4 weeks), and (5 mg twice daily + 480 mg every 4 weeks).

##### Toxicity and Pharmacokinetic Data from Public Data Sources

**FIGURE 3:**
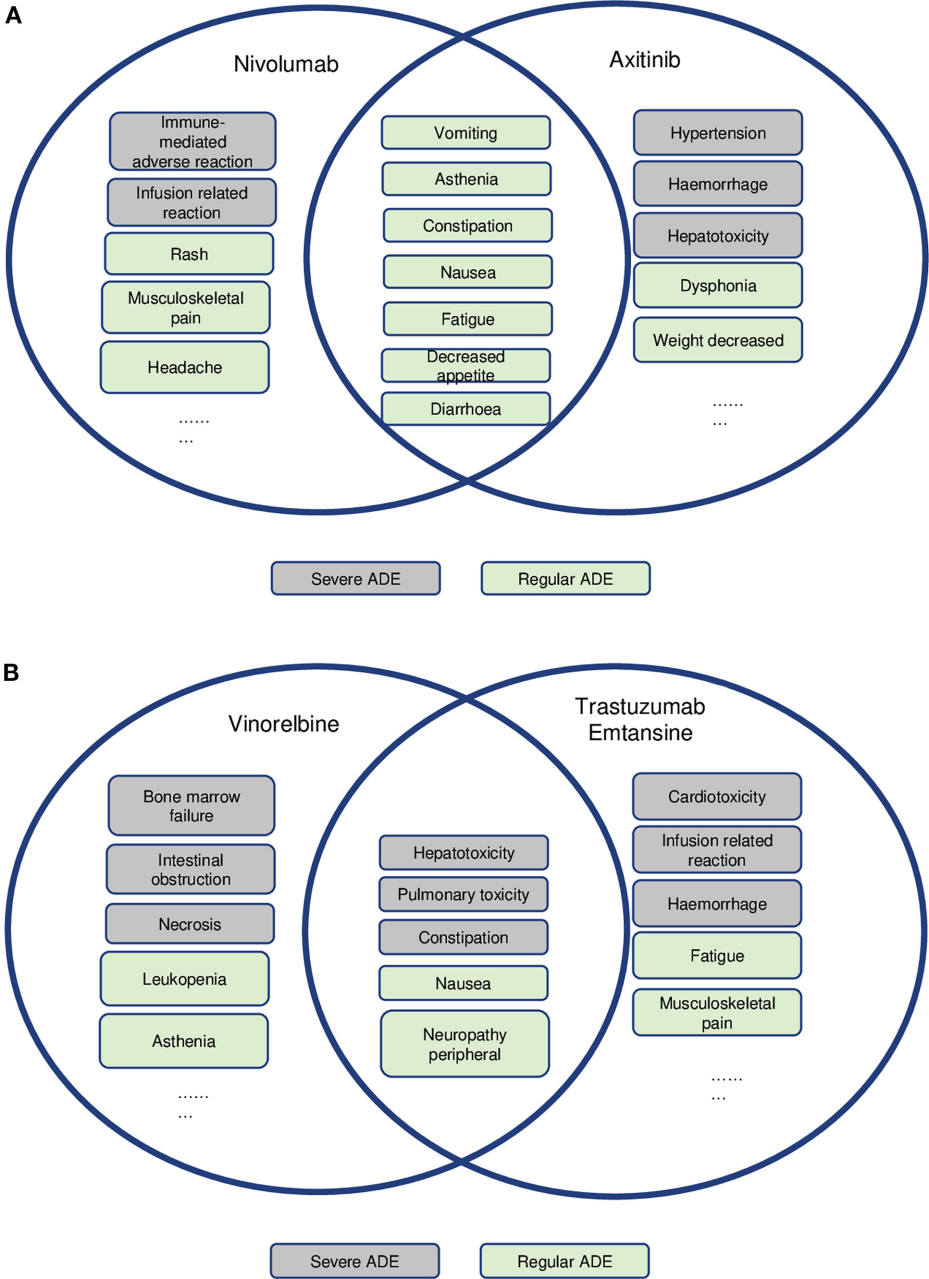
Overlap of toxicities and their severity among drug combinations in two reported cases. **A,** Overlapping toxicities of nivolumab and axitinib. **B,** Overlapping toxicities of vinorelbine and trastuzumab emtansine.

The FDA first approved axitinib, a small molecule and tyrosine kinase inhibitor, in 2011 for the treatment of advanced renal cell carcinoma. The most common (≥20%) adverse reactions include diarrhea, hypertension, fatigue, decreased appetite, nausea, dysphonia, palmar-plantar erythrodysesthesia syndrome, weight loss, vomiting, asthenia, and constipation. Nivolumab, a biologic antibody and PD-1 inhibitor, was approved in 2014 for the treatment of various types of cancer. The combination's pharmacokinetic and toxicity evidence and strength annotation was summarized in Table 2 and Fig. 3. Our review indicated that the toxicity profile of nivolumab (Fig. 3A) attributed most nivolumab-induced ADEs to immune-related ADEs, while axitinib could probably induce cardiovascular and hemal events. These two drugs have only one severe overlapping ADE (embryo-fetal toxicity). A review of the literature indicated MTDs of 5 mg twice daily for axitinib and 480 mg for nivolumab (11). More details regarding this trial are provided in the Supplementary Data S3. Therefore, no pharmacokinetic interactions were expected in this study.

**TABLE 2:** Evidence from prior investigation data and corresponding strength annotation for two cases

	PK data			Toxicity data			
Combination	Evidence	Source	Strength annotation	Evidence	Source	Strength annotation	Class
Nivolumab/ axitinib	Axitinib is metabolized primarily by CYP3A4/5 and, to a lesser extent, by CYP1A2, CYP2C19, and UGT1A1 (14), whereas nivolumab is mostly degraded in targeted tissues other than the liver (15).	Literature	PK-DDI I(-)	These two drugs have only one overlapping ADE (embryo-fetal toxicity).	Drug Label	Tox-III	III
Vinorelbine/ trastuzumab emtansine	Vinorelbine is primarily metabolized through CYP450s (16), whereas trastuzumab is primarily degraded in targeted tissues other than the liver (17).	Literature	PK-DDI I(-)	These two drugs have several serve overlapping toxicities including hepatotoxicity, pulmonary toxicity, thrombocytopenia, and congenital disabilities.	Drug Label	Tox-III	III

##### Phase I Trial Redesign

This phase I study could be redesigned as a phase IB study to start at a 5 mg maximum dose of axitinib with dose reduction to 3 and 2 mg if needed and maintenance of a 3 mg/kg dose of nivolumab based on guidelines. A dose of 5 mg for axitinib and 3 mg/kg for nivolumab could potentially achieve an optimum combination dose with therapeutic effects while ensuring patient safety. The period for evaluating DLT remained the same for the first cycle. However, the definition of DLT could be modified to include any grade 3 or higher non-hematologic toxicity, any grade 4 hematologic toxicity that does not resolve within 48 hours, any grade 2 diarrhea or colitis that does not resolve within 48 hours with supportive care, any grade 3 colitis or diarrhea, or any grade 3 hypertension. This would allow for an expected DLT rate of 25% at the MTD, based on the high rate of hypertension with axitinib, which would not be expected to be exacerbated by nivolumab. With this additional preliminary information, adaptive statistical methods could be applied to redesign this phase I combination trial and minimize patient exposure to subtherapeutic dose levels.

#### Case 2, Vinorelbine and Trastuzumab Emtansine

##### Original Study Design

In 2016, previously treated patients with HER2-positive metastatic breast cancer participated in a phase IA trial of vinorelbine and trastuzumab emtansine (T-DM1; NCT02658084), in which trastuzumab was administered at a consistent dose of 3.6 mg/kg, and vinorelbine was administered at five dose levels from 20 to 30 mg/m^2^.

##### Toxicity and Pharmacokinetic Data from Public Data Sources

Vinorelbine is a small molecule and antimitotic chemotherapy agent approved by the FDA in 1994 for non–small cell lung cancer, and T-DM1 is an antibody–drug conjugate and anti-HER2 therapy approved in 2013 for metastatic breast cancer. The most common ADEs observed with T-DM1 were nausea, fatigue, musculoskeletal pain, thrombocytopenia, increased transaminase levels, headache, and constipation. The most common severe ADEs are thrombocytopenia, increased transaminase levels, anemia, hypokalemia, peripheral neuropathy, and fatigue. Moreover, the results of a phase I study of vinorelbine in patients with carcinomas, lymphomas, and chronic myeloid leukemia showed an MTD of 35 mg/m^2^ weekly if it was administered as monotherapy (12), and granulocytopenia was noted as a DLT. The results of another phase I study of vinorelbine in metastatic breast cancer showed an MTD of 40 mg/m^2^, and a subsequent phase II study determined it to be 35 mg/m^2^ (13). The combination's pharmacokinetics and toxicity evidence and strength annotation was summarized in Table 2 and Fig. 3. A review of their toxicity profiles (Fig. 3B) showed similarities, including hepatotoxicity, pulmonary toxicity, thrombocytopenia, and congenital disabilities. Among the shared toxicities, drug labels designate hepatotoxicity and congenital disabilities as severe ADEs. More details regarding this trial are provided in Supplementary Data S3. No pharmacokinetic interactions are expected between these two drugs.

##### Phase I Trial Redesign

On the basis of the evidence regarding pharmacokinetic DDI and toxicities, we believe that the use of this combination should be avoided because of the similar toxicity patterns of the individual drugs. Otherwise, this combination could be evaluated carefully in clinical trials using a standard 3+3 design with a low starting dose. According to ClinicalTrials.gov, NCT02658084 was terminated because of low accrual and toxicity concerns, including cardiac, gastrointestinal, metabolism, and nutritional disorders, as well as some general disorders.

## Discussion

Multiple therapeutic designs targeting various cancer pathways or regulation networks have yielded more effective results with respect to anticancer effects than designs utilizing single-agent regimens. Thus, the number of preclinical and clinical studies on such drug combinations has increased to accelerate the translation of investigative drugs to the clinical setting. However, many studies have indicated the failure of many drug combinations for cancer therapies during phase I trials (1). Incorporating all available knowledge regarding the properties of both single drugs and their combinations in the design of phase I trials might overcome this high failure rate. The use of prior lessons might facilitate the selection of appropriate doses and schedules to avoid severe or life-threatening ADEs, thereby maximizing patient safety and developmental efficiency.

In this study, we reviewed several publicly available databases that provide extensive information regarding the pharmacokinetic and toxicity profiles of individual drugs and drug combinations. The FM, DIDB, and UCSF-Transportal databases serve as repositories of quantitative data regarding metabolic enzymes and transportation that are critical in measuring pharmacokinetic DDIs, and DailyMed serves as a resource for critical drug toxicity data. Moreover, several drug combination databases have collected experimental and clinical evidence related to DDIs.

However, some essential information, such as MTD and DLT, remains widely scattered in the published literature, and unstructured formatting limits accessibility and facilities for computerized integration and analysis. Storage of drug labels in their original format in the DailyMed database, for example, only allows the labels to be read. The most critical limitation of these valuable data sources is that they are not well integrated in either the user or data layer.

In this study, we designed a classification approach that could assist in designing drug combination phase I clinical trial by synthesis of prior pharmacokinetic DDI and toxicity evidence. Our study also demonstrated the feasibility and utility of incorporating as much knowledge as possible from prior investigations to assist in the design of new phase I trials of drug combinations. Among the 423 such investigations for cancer therapies reported in ClinicalTrials.gov from January 1 to December 31, 2018, we identified 268 unique two-drug combinations, and more than 70% of these included at least partial prior knowledge of pharmacokinetics or toxicity. In addition, four combinations were evaluated under other conditions or patient populations. Our study also illustrated that careful review of all these data sources might have led to a better design of some earlier phase I studies.

Examination of prior knowledge regarding the pharmacokinetic mechanisms of nivolumab and axitinib revealed that no pharmacokinetic DDI might be expected from their combined use. Furthermore, the toxicity profiles indicated no overlapping toxicities. Thus, the reviewing physician considered a phase IB study with alternative dose deescalation based on additional information as a preferable approach. In addition, the reviewing statistician felt that the use of adaptive statistical methods to accelerate the trial process would minimize patient exposure to subtherapeutic dose levels.

Synthesis of prior knowledge regarding vinorelbine and T-DM1 revealed significant overlapping toxicities between them, suggesting that they should not be used in combination to treat patients, or that such use should be carefully evaluated in clinical trials.

Our study has several limitations. We manually reviewed several data sources to integrate valuable information regarding the design of previous phase I trials of drug combinations. We collected information already processed, such as normalized drug names, and retrieved information from PubMed and other sources using keyword searches. More critical information is needed to assist in the trial design. For example, we extracted ADEs from drug labels and mapped them to MedDRA PTs, but some error might occur during the mapping procedure. Moreover, incorporating evidence regarding the severity of ADEs could provide more stratified and detailed information to help define accurate DLTs. Future work should include massive and automatic retrieval and collection of information using machine-learning technology to expand the current drug combination knowledge base. Such a deep integration of accumulated prior knowledge regarding the pharmacokinetics, toxicity, and pharmacology of single drugs and their combinations and a user-friendly design of that information base would promote the application of this knowledge to the better design of phase I trials to treat cancer.

## Supplementary Material

Supporting Infomation S1 Supporting Infomation S1 provides an overview of data source for drug combination Phase I trial design

Supporting Information S2 Supporting Information S2 provides classification results offor cancer drug combination phase I clinical trials in clinicaltrials.gov (01 January through 31 December 2018)using the designed approach in the paper.

Supporting Information S3 Supporting Information S3 provides original study design of two case studies.

## References

[1] Wong CH , Siah KW, Lo AW. Estimation of clinical trial success rates and related parameters. Biostatistics 2019;20:273–86. 29394327 10.1093/biostatistics/kxx069 PMC6409418

[2] Le Tourneau C , Lee JJ, Siu LL. Dose escalation methods in phase I cancer clinical trials. J Natl Cancer Inst 2009;101:708–20. 19436029 10.1093/jnci/djp079 PMC2684552

[3] Friedman LM , Furberg CD, DeMets DL. Fundamentals of clinical trials. New York, NY: Springer; 2010.

[4] Chow S-C . Adaptive clinical trial design. Annu Rev Med 2014;65:405–15. 24422576 10.1146/annurev-med-092012-112310

[5] van Brummelen EMJ , Huitema ADR, van Werkhoven E, Beijnen JH, Schellens JHM. The performance of model-based versus rule-based phase I clinical trials in oncology: A quantitative comparison of the performance of model-based versus rule-based phase I trials with molecularly targeted anticancer drugs over the last 2 years. J Pharmacokinet Pharmacodyn 2016;43:235–42. 26960536 10.1007/s10928-016-9466-0

[6] Zhou H , Yuan Y, Nie L. Accuracy, safety, and reliability of novel phase I designs—response. Clin Cancer Res 2018;24:5483–4. 30385656 10.1158/1078-0432.CCR-18-2677

[7] Wishart DS , Feunang YD, Guo AC, Lo EJ, Marcu A, Grant JR, . DrugBank 5.0: a major update to the DrugBank database for 2018. Nucleic Acids Res 2018;46:D1074–82. 29126136 10.1093/nar/gkx1037 PMC5753335

[8] Kuhn M , Letunic I, Jensen LJ, Bork P. The SIDER database of drugs and side effects. Nucleic Acids Res 2016;44:D1075–9. 26481350 10.1093/nar/gkv1075 PMC4702794

[9] Hua L , Chiang CW, Cong W, Li J, Wang X, Cheng L, . The cancer drug fraction of metabolism database. CPT Pharmacometrics Syst Pharmacol 2019;8:511–9. 31206254 10.1002/psp4.12417 PMC6656935

[10] Morrissey KM , Wen CC, Johns SJ, Zhang L, Huang SM, GKM. The UCSF-FDA TransPortal: a public drug transporter database. Clin Pharmacol Ther 2012;92:545–6. 23085876 10.1038/clpt.2012.44 PMC3974775

[11] Gibney GT , Kudchadkar RR, DeConti RC, Thebeau MS, Czupryn MP, Tetteh L, . Safety, correlative markers, and clinical results of adjuvant nivolumab in combination with vaccine in resected high-risk metastatic melanoma. Clin Cancer Res 2015;21:712–20. 25524312 10.1158/1078-0432.CCR-14-2468 PMC4620684

[12] Mathé G , Reizenstein P. Phase I pharmacologic study of a new Vinca alkaloid: navelbine. Cancer Lett 1985;27:285–93. 4016723 10.1016/0304-3835(85)90186-7

[13] Sorio R , Robieux I, Galligioni E, Freschi A, Colussi AM, Crivellari D, . Pharmacokinetics and tolerance of vinorelbine in elderly patients with metastatic breast cancer. Eur J Cancer 1997;33:301–3. 9135505 10.1016/s0959-8049(96)00426-1

[14] Zientek MA , Goosen TC, Tseng E, Lin J, Bauman JN, Walker GS, . *In**vitro* kinetic characterization of axitinib metabolism. Drug Metab Dispos 2016;44:102–14. 26512042 10.1124/dmd.115.065615

[15] Feld E , Horn L. Emerging role of nivolumab in the management of patients with non-small-cell lung cancer: current data and future perspectives. Onco Targets Ther 2017;10:3697–708. 28769573 10.2147/OTT.S97903 PMC5533488

[16] Kajita J , Kuwabara T, Kobayashi H, Kobayashi S. CYP3A4 is mainly responsibile for the metabolism of a new vinca alkaloid, vinorelbine, in human liver microsomes. Drug Metab Dispos 2000;28:1121–7. 10950859

[17] Boekhout AH , Beijnen JH, Schellens JHM. Trastuzumab. Oncologist 2011;16:800–10. 21632460 10.1634/theoncologist.2010-0035 PMC3228213

