# Supplementary material for: An Informatics Bridge to Improve the Design and Efficiency of Phase I Clinical Trials for Anticancer Drug Combinations
Source: Cancer Res Commun. 2022 Sep 6;2(9):929–36. doi: 10.1158/2767-9764.CRC-22-0160 (PMC10010310; doi:10.1158/2767-9764.CRC-22-0160)
Supplement: Supporting Infomation S1 — provides an overview of data source for drug combination Phase I trial design [file crc-22-0160-s01.pdf]

Data source overview for drug combination Phase I trial design

Proper drug combination Phase I trial design requires the acquisition and thorough evaluation of all available evidence and knowledge regarding MTDs of individual drug and/or in combination, single drug pharmacokinetics and their interactions, dose-limiting toxicity (DLT) for single drugs, and overlapping toxicities among them. These evidences and prior knowledge can be adequately accessed from different data sources. However, PK and toxicity data are generally reported from different sources. The following is a comprehensive PK and toxicity data review.

Drug databases with comprehensive drug data The DrugBank(1) is a well-known comprehensive database for 13,571 drugs developed by the Wishart Research Group. It provides information for potential DDI evidences for the investigational drug combinations. However, the qualitative DDI descriptions in DrugBank does not help us in understanding of the DDI magnitude and mechanisms underlying the interactions. The toxicity data are limited. Drugs.com(2) is a commercial database that provides curated texted data information for more than 24,000 drugs. It includes the toxicity data extracted from drug labels and DDI data reported in clinical studies, such as the ratio of the area under the plasma concentration-time curve (AUCR).

Drug pharmacokinetics data sources include *Goodman & Gilman's The Pharmacological Basis of Therapeutics(3),* Cancer Drug Fraction of Metabolism Database (FM database)(4), Drug-interaction Database (DIDB) of the University of Washington (UW)(5), TransPortal database of the University of California, San Francisco (UCSF) in partnership with the FDA(6), and Transformer database(7), developed by the Structural Bioinformatics Group of the Charité Institute for Physiology in Berlin. These data sources collect direct evidence of PK DDI, such as the observed AUCR from clinical studies or in vitro experiments. Goodman & Gilman's book, for example, contains PK parameters of 278 on-market drugs. The FM database currently provides the quantitative contribution of individual CYP enzymes from published in *vitro* studies for 42 cancer drugs. UW's DIDB has manually curated in vitro kinetic parameters, inhibition and

induction values and in vitro-to-in vivo prediction ratios for 2,125 substrates, 4,433 inhibitors, 225 activators, and 1,500 inducers. The UCSF-FDA TransPortal database integrates information regarding transporter expression, localization, substrates, inhibitors for 482 substrates, 866 inhibitors, and 48 clinical transporter-based DDIs. Transformer contains 4,400 interactions of phase I and II enzymes and 1,158 drug-transporter interactions involving 2,800 drugs, prodrugs, and some herbs.

Drug-toxicity data sources The DailyMed website(8), maintained by the National Library of Medicine, provides up-to-date information, such as drug toxicity and the corresponding frequency, patient population and size from 125,851 drug labels. The Side Effect Resource (SIDER)(9) organizes drug-toxicity data extracted from drug labels and other public resources in a structured way, mapping ADEs to the preferred terms (PT) of the *Medical Dictionary of Regulatory Activities* (MedDRA). ClinicalTrials.gov(10), also maintained by the National Library of Medicine, is a clinical trial registry that contains more than 345,570 research studies worldwide. It provides toxicity data, including ADE frequencies, MTD and DLT, and the disease populations. The FDA's Adverse Event Reporting System (FAERS)(11), a post-marketing pharmacovigilance database, serves as well as a clinical database for toxicity data. FAERS allows review of the toxicity properties of an individual drug or drug combination in a large patient population, but it reports only ADEs and not their frequency. OFFSIDE and TWOSIDES(12), are two databases, in which single drug and drug combination data are derived from FAERS. Besides these databases, the primary drug toxicity data, including clinical trial results, are widely scattered over the scientific literature including publications in PubMed, abstracts in the American Society of Clinical Oncology annual meetings and the American Association for Cancer Research annual meetings. The MTDs, DLTs, ADE frequency, and patient population can be found from them.

Reference:

1. Wishart DS, Feunang YD, Guo AC, Lo EJ, Marcu A, Grant JR*, et al.* DrugBank 5.0: a major update to the DrugBank database for 2018. *Nucleic Acids Research* 2018;**46**(D1):D1074-D82 doi 10.1093/nar/gkx1037.
2. Limited D. Drugs.com. 2020.
3. Brunton LL, Hilal-Dandan R, Knollmann BC, editors. Goodman & Gilman's The pharmacological basis of therapeutics. Thirteenth edition ed. New York; Chicago; San Francisco: McGraw Hill Education; 2018. 1419 p.
4. Hua L, Chiang CW, Cong W, Li J, Wang X, Cheng L*, et al.* The Cancer Drug Fraction of Metabolism Database. *CPT: Pharmacometrics & Systems Pharmacology* 2019;**8**(7):511–9 doi 10.1002/psp4.12417.
5. U W. UW Drug Interaction Solutions. <www.druginteractionsolutions.org/>.
6. Morrissey KM, Wen CC, Johns SJ, Zhang L, Huang S-M, Giacomini KM. The UCSF-FDA TransPortal: A Public Drug Transporter Database. *Clinical Pharmacology & Therapeutics* 2012;**92**(5):545–6 doi 10.1038/clpt.2012.44.
7. Hoffmann MF, Preissner SC, Nickel J, Dunkel M, Preissner R, Preissner S. The Transformer database: biotransformation of xenobiotics. *Nucleic Acids Research* 2014;**42**(D1):D1113-D7 doi 10.1093/nar/gkt1246.
8. MEDICINE USNLO. 8/26. DailyMed. <https://dailymed.nlm.nih.gov/dailymed/>. Accessed 2020 8/26.
9. Kuhn M, Letunic I, Jensen LJ, Bork P. The SIDER database of drugs and side effects. *Nucleic Acids Res* 2016;**44**(D1):D1075-9 doi 10.1093/nar/gkv1075.
10. MEDICINE USNLO. ClinicITrails.gov. <https://clinicaltrials.gov/>.
11. Administration USFaD. FDA Adverse Event Reporting System (FAERS). <https://fis.fda.gov/extensions/FPD-QDE-FAERS/FPD-QDE-FAERS.html>.
12. Tatonetti NP, Ye PP, Daneshjou R, Altman RB. Data-Driven Prediction of Drug Effects and Interactions. *Science Translational Medicine* 2012;**4**(125):125ra31-ra31 doi 10.1126/scitranslmed.3003377.
