# Supplementary material for: An Informatics Bridge to Improve the Design and Efficiency of Phase I Clinical Trials for Anticancer Drug Combinations
Source: Cancer Res Commun. 2022 Sep 6;2(9):929–36. doi: 10.1158/2767-9764.CRC-22-0160 (PMC10010310; doi:10.1158/2767-9764.CRC-22-0160)
Supplement: Supporting Information S3 — provides original study design of two case studies. [file crc-22-0160-s03.pdf]

S3: Original detailed study design of two special cases

nivolumab and axitinib Six to twelve patients were assessed during the original Phase I trial, which utilized a standard “3+3” design to evaluate three doses of axitinib along with a standard fixed dose of nivolumab. The starting 3-mg dose of axitinib could be escalated to 5 mg or de-escalated to 2 mg with the 480-mg dose of nivolumab based on dose-limiting toxicities. DLTs were defined as severe hematologic ADEs, including neutropenia, febrile neutropenia, neutropenic infection, and thrombocytopenia with or without bleeding, and severe non-hematologic ADEs, including nausea, vomiting, diarrhea, iritis, episcleritis, eye pain, blurred vision, and hypertension*.* All the DLTs occurred within the 28-day assessment window.

vinorelbine and trastuzumab emtansine In the original design of the protocol, the Phase I portion employed a standard 3+3 dose escalation/de-escalation scheme utilizing five dose levels of Vinorelbine and a fixed dose of trastuzumab. The starting dose is at Vinorelbine 22.5 mg/m^2 and trastuzumab 3.6 mg/kg. Dose-limiting toxicities were defined as severe non-hematologic toxicities that limit the ability of the patient to tolerate treatment on schedule, neuropathy, neutropenia, elevated levels of liver function tests, and left ventricular systolic dysfunction, and these were to be assessed over the first two cycles.
